# Age-invariant benefits of spatiotemporal predictions amidst distraction during dynamic visual search

**DOI:** 10.1038/s41598-025-01796-4

**Published:** 2025-05-16

**Authors:** Nir Shalev, Sage Boettcher, Anna C. Nobre

**Affiliations:** 1 Department of Gerontology, Faculty of Social Welfare and Health Sciences, University of Haifa, Haifa, IL Israel; 2 The Institute of Information Processing and Decision Making (IIPDM), University of Haifa, Haifa, IL Israel; 3 Department of Experimental Psychology, University of Oxford, Oxford, UK; 4 Oxford Centre for Human Brain Activity, Wellcome Centre for Integrative Neuroimaging, Department of Psychiatry, University of Oxford, Oxford, UK; 5 Wu Tsai Institute, Yale University, New Haven, USA

**Keywords:** Visual search, Lifespan, Ageing, Attention, Cognitive ageing, Cognitive neuroscience, Learning and memory, Visual system, Human behaviour

## Abstract

Visual search tasks are widely used to study attention amidst distraction, often revealing age-related differences. Research shows older adults typically exhibit poorer performance and greater sensitivity to distraction, reflecting declines in goal-driven attention. However, traditional search tasks are static and fail to capture the challenges and opportunities in natural environments, which include predictive structures within extended contexts. We designed a search variation where targets and distractors compete over time and embedded spatiotemporal regularities afford prediction-led guidance of attention. Critically, we manipulated the number of distractors to chart how benefits of expectations and deficits from distraction varied with age. Younger and older adults searched for multiple targets as they faded in and out of the display while varying the number of distracting elements between trials. Half the targets appeared at the same time and approximate locations and could be predicted. While we found evidence for decrement and elevated sensitivity to distraction with increasing age, benefits from predictions occurred in all groups. Interestingly, regardless of age, effects of predictions were only significant during periods of high distraction. This work extends our understanding of attention control through ageing to dynamic settings and indicates a dissociation between goal-directed and learning-driven attentional guidance.

## Introduction

The ability to select stimuli that are relevant to our goal amidst competing distraction is a pillar of adaptive behaviour. In experimental settings, this can be studied using the Visual Search task^1,2^. In standard visual search tasks, participants search for a predefined visual stimulus (“target”) that appears among distracting stimuli in a static array. This simple experimental setup can reveal the factors that determine search efficiency, such as the number of distractors, their relative salience, or similarity to the target (e.g.,)^3,4^. These experiments also highlight factors that guide search. Visually salient items automatically attract sensory processing (e.g.,)^5^; Task-relevant representations of target features or locations facilitate target identification (e.g.,)^6,7^; and memories of target-distractor attributes and configurations influence performance in multiple ways (e.g.,)^8–10^.

As the cognitive system continuously changes throughout the lifespan, so does our ability to guide attention. Older adults perform relatively poorly in visual search tasks compared to younger adults (e.g.,)^11–13^. This decline is attributed to various factors, including age-related changes specifically related to cognitive control^14,15^ as well as general changes in perceptual abilities^16^ and reduced processing speed^17^. Yet, it is notable that age-related decline in search performance does not occur in all contexts. For instance, it has been proposed that the fundamental mechanisms of spatial attention orienting remain relatively preserved with age, while the observed declines are more likely attributed to interactions with higher-order cognitive functions^18^. In studies directly involving visual search, differences across age groups diminish in task designs in which prior experience can guide search^19–22^. When observers can anticipate the location or identity of targets based on memory, search performance can improve regardless of age^23,24^, even to a comparable level between younger and older adults^25^.

We recently developed a Dynamic Visual Search task (DVS) in which participants search for multiple targets among competing distractors within temporally extended and dynamic contexts^26–29^. The task design captures the common experience outside of the laboratory, where targets and distractors compete for attention in the flux of the environment. For example, when driving across a busy intersection, the cars travelling in the same direction as us can be safely ignored, while the cars coming towards us must be carefully monitored. The DVS task was designed to investigate how experience-driven spatiotemporal predictions guide attention in dynamic contexts. In the DVS, targets and distractors fade in and out of the display at various locations and onsets during temporally extended trials. Participants must find eight visual targets in each trial. Critically, four targets occur predictably, appearing at the exact time and approximate location across trials. The other four targets appear unpredictably, occurring at any time and location. As a result, participants can learn to predict the occurrence of half of the targets.

By comparing hit rate and response speed to predictable vs. random targets, we observed that neurotypical young adults benefit from spatiotemporal regularities^26–28^. Testing children and older adults showed comparable effects^22,30,31^. Interestingly, applying the DVS in a developmental context, we showed a functional dissociation between overall search performance and prediction-based benefits. Children and older adults performed worse than young adults in overall search performance. Yet, their ability to benefit from spatiotemporal predictions was robust and unimpaired.

In the present study, we asked whether and how the number of distractors in a search display interacted with the benefits of spatiotemporal predictions in younger and older adults. The design enabled us to probe several important facets of visual search between the age groups. Do benefits of spatiotemporal predictions scale with the sensory demands of individuating targets among distractors? Do interactive effects change between the age groups? And finally, do the usual disproportionate costs of high distraction in older adults documented in static search also occur for dynamic and temporally extended visual search?

Our task required participants to identify spatiotemporally predictable and unpredictable targets among varying numbers of similar distracting stimuli. We tested two hundred and forty individuals in two age groups (120 in each group of younger and older adults). To foreshadow our results, we discovered a similar pattern of adaptive utilization of spatiotemporal predictions depending on the number of distractors for both age groups. Predictions conferred significant benefit as the number of distractors increased, to a similar extent for young and old participant. Despite benefitting from predictions, older participants nevertheless had worse search performance in general and showed greater costs with more distraction.

## Results

### Task performance

**Fig. 1:**
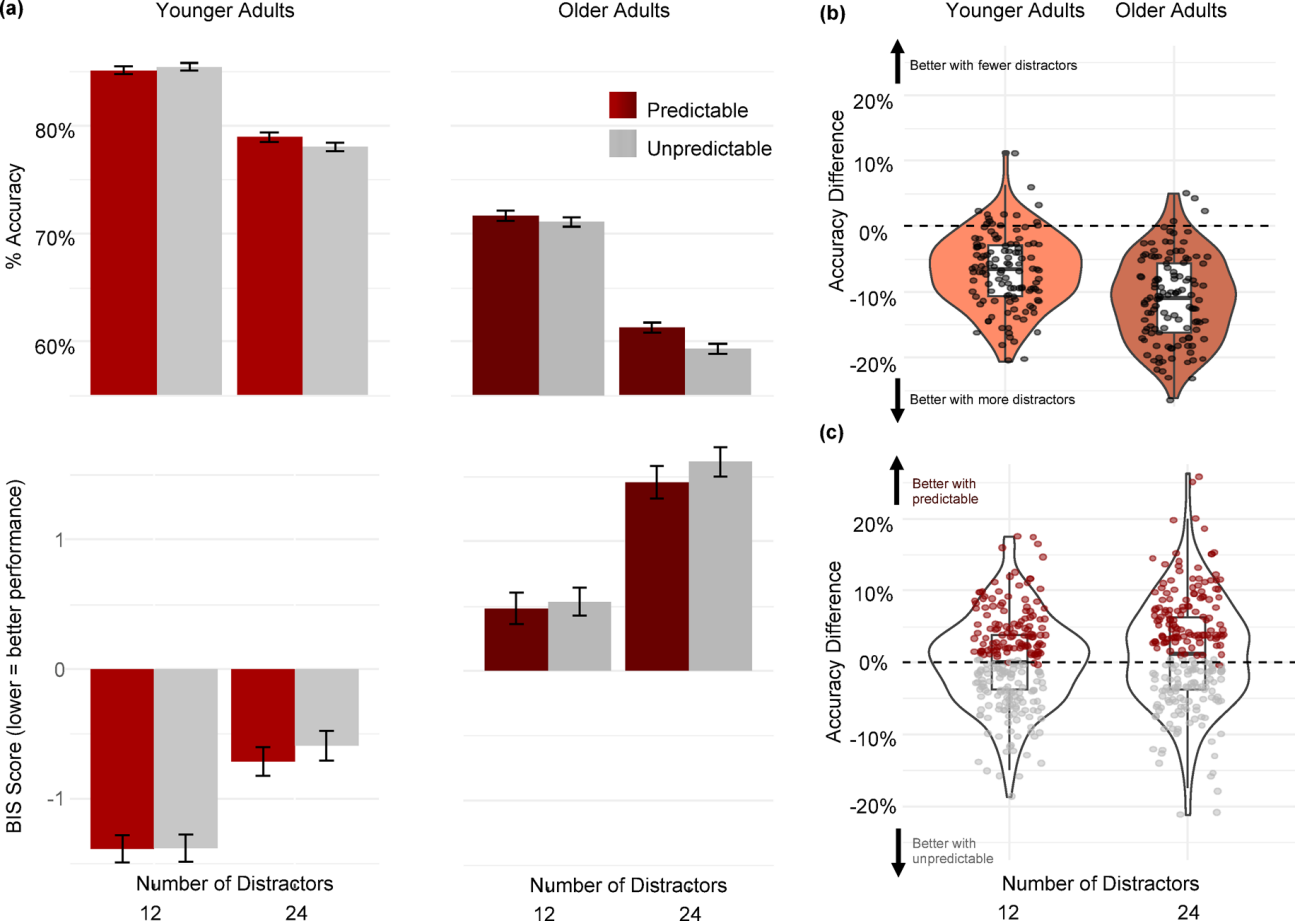
**(a)** Task performance grouped by experimental conditions (Age × Target Predictability × Number of Distractors). In the upper two figures, the Y-axis shows the percentage of identified targets (higher scores mean better performance). The lower figures show in the Y-axis the BIS score, combining accuracy and reaction times (lower scores mean better performance). The left panels represent younger adults and the right represents older adults, with 120 participants per group. The X-axis within each panel indicates the Number of Distractors (12 distractors vs. 24 distractors). Coloured bars denote target predictability: red for predictable targets and grey for random targets; **(b)** The difference in accuracy between high and low distraction as a function of Age Group. The significant interaction between Number of Distractors × Age Group was driven by greater behavioural costs of adding visual distractors in the older adult group. Values below “0” (marked by the horizontal dashed line) indicate fewer targets found when they appeared among a higher number of distractors. Violin plots show the density and distribution of the difference between the two conditions. Individual dots represent participants; **(c)** The difference in accuracy between predictable and unpredictable targets as a function of Number of Distractors. The significant interaction between Number of Distractors × Predictability was driven by finding more predictable targets only when the distraction load was high. Values above “0” (marked by the horizontal dashed line) indicate more predictable targets found compared to unpredictable ones. Violin plots show the density and distribution of the difference between the two conditions. Individual dots represent participants.

Mean hit rates across the experimental conditions appear in Fig. 1. Analysis of the hit rate revealed main effects of Age Group (F(1,238) = 109.61, *p* <.001; η2_G_ = 0.2), Predictability (F(1,238) = 5.11, *p* =.02; η2_G_ = 0.009), and Number of Distractors (F(1,238) = 514.94, *p* <.001; η2_G_ = 0.11). Specifically, younger adults found more targets than older adults (younger: 81% vs. older: 65%), predictable targets were overall found more often than unpredictable targets (predictable: 74% vs. unpredictable: 73%), and participants found more targets when presenting 12 distractors compared to when presenting 24 distractors (12 distractors: 78% vs. 24 distractors: 69%).Two-way interactions were significant between Age Group X Number of Distractors (F(1,238) = 29.45, *p* <.001; η2_G_ = 0.0007) and Predictability X Number of Distractors (F(1,238) = 5.02, *p* =.02; η2_G_ = 0.0006). There were no other significant effects (all p’s > 0.15).

We interpreted the significant two-way interactions of Age X Number of Distractors using post hoc analyses. First, we compared accuracy between the low and high number of distractors within each age group separately. Increased distraction worsened performance in both groups: younger adults (β = −0.068, SE = 0.004, df = 714, t ratio = −13.873, *p* <.001) and older adults (β = −0.111, SE = 0.004, df = 714, t ratio = −22.594, *p* <.001). Therefore, our manipulation effectively influenced performance in both groups and the interaction effect instead appears to be driven by a difference in effect sizes, which were smaller for younger adults (Cohen’s d = −1.27, SE = 0.095, df = 314, 95% CI [−1.46, −1.08]) compared to older adults (Cohen’s d = −2.06, SE = 0.102, df = 314, 95% CI [−2.27, −1.86]). Accordingly, older adults may pay a greater behavioural cost between the two conditions of the Number of Distractors, compared to young adults.

To determine whether the difference in the effect size of Number of Distractors was statistically significant, we used the emmeans package in R to calculate the estimated marginal means for each combination of age group and distraction level. We then conducted pairwise comparisons between the different levels of distraction within each age group. Finally, we compared these differences to test if the effect of distraction on accuracy varied between the two age groups. The results indicated a statistically significant difference between the groups (β = −0.043, SE = 0.006, df = 714, t ratio = 6.167, *p* <.001).

A second post hoc analysis interpreted the interaction of Number of Distractors X Prediction. We compared the different levels of predictability for each level of distraction and discovered that predictions benefitted behaviour significantly when the distraction load was high. Under high distraction load, accuracy declined significantly (β =−0.01; SE = 0.004; df = 714; t.ratio = −2.932; *p* =.003). In contrast, under a low distraction load, the effect of predictability was not significant (β = −0.01; SE = 0.004; df = 714; t.ratio =−0.287; *p* =.77).

We supplemented our primary accuracy analysis with a comparison of mean reaction times and the mean Balanced Integration Score (BIS). The BIS is a combined score that reflects both speed and accuracy in the dynamic visual-search task, calculated from standardised mean values based on each participant’s mean.

When comparing reaction times, we detected main effects of the Group factor (F(1,238) = 24.77, *p* <.001; η2_G_ = 0.002), Number of Distractors (F(1,238) = 261.52, *p* <.001; η2_G_=. 327), and Predictability (F(1,238) = 5.06, *p* =.025; η2_G_ = 0.007). All other p’s were > 0.15. Accordingly, older adults were slower than younger adults in their reaction times, adding more distractor led to slower responses, and predictable targets were found faster.

When analysing the BIS, ss with accuracy, there was a main effects of Age Group (F(1,238) = 179.94, *p* <.001; η2_G_ = 0.40), Predictability (F(1,238) = 7.15, *p* =.007; η2_G_ = 0.001), and Number of Distractors (F(1,238) = 902.58, *p* <.001; η2_G_ = 0.11). Young adults were better overall than older adults, participants were better at finding predictable compared to unpredictable targets, and overall they performed better when there were 12 distractors compared to 24. The BIS results also replicated the two-way interactions between Age Group X Number of Distractors (F(1,238) = 25.19, *p* <.003; η2_G_ = 0.11) and Predictability X Number of Distractors (F(1,238) = 5.3, *p* <.02; η2_G_ = 0.0004). There were no other significant effects. Post hoc analyses clarified the interactions. Comparing Number of Distractors effects for each group, showed significant effects for both younger (β = 0.734; SE = 0.04; df = 714; t.ratio = 18.233; *p* <.001) and older (β = 1.028; SE = 0.04; df = 714; t.ratio = 25.548; *p* <.001) adults. The interaction was likely to be driven by a smaller effect size for young adults (Cohen’s d = 1.55; SE = 0.099; df = 255; 95%CI[1.47;1.86]) compared to older adults (Cohen’s d = 2.33; SE = 0.106; df = 255; 95%CI[2.12;1.54]). The data are depicted in Fig. #2.

As with accuracy, we tested whether the difference in performance between levels of distraction condition differed between groups by comparing the t-ratios between two contrasts. We used the emmeans package in R to calculate the estimated marginal means for each combination of age group and distraction level. Next, we conducted a pairwise comparisons between different levels of distraction within each age group. Finally, we compared the differences we modelled to test if the effect of distraction on accuracy was different between the two age groups. The results indicated a statistically significant difference (β = −294; SE = 0.057; df = 714; t.ratio = −5.173; *p* <.001).

A second posthoc analysis compared the different levels of predictability for each level of distraction. Predictability significantly improved performance under high Number of Distractors (β = 0.14; SE = 0.04; df = 714; t.ratio = 3.467; *p* <.001) with a small-medium effect size (Cohen’s d = 0.31; SE = 0.09; df = 289; 95%CI[0.136;0.497]). Under low distraction load, the effect of predictability was not significant (β = 0.03; SE = 0.04; df = 714; t.ratio = 0.746; *p* =.45).

Finally, two of our statistical models (analysing Accuracy and BIS) relied on Null Hypothesis Statistical Testing, which resulted in a non-significant interaction between Age Group and Predictability. To further interpret this null effect, we calculated the difference in performance between predictable and unpredictable targets for each participant and compared the mean scores between the two groups using a Bayesian t-test. First, we focused on accuracy. The test yielded a Bayes Factor of 0.37, indicating that the observed data are approximately 2.7 times more likely under the null hypothesis than under the alternative hypothesis. This suggests moderate evidence against a difference in predictions between the age groups. We applied the same approach to the BIS score, which includes response speed measures. This analysis produced a Bayes Factor of 0.18, meaning that the data are about 5.7 times more likely to have occurred under the null hypothesis than under the alternative hypothesis. Together, these findings provide moderate to strong evidence supporting the null hypothesis, suggesting that there is likely no substantial difference between the age groups in terms of predictions.

## Discussion

Compared to younger adults, older adults had worse performance on the dynamic visual search task and were more impaired by distraction. Yet, they benefited from spatiotemporal predictions for identifying targets to the same extent. These results thus highlight a clear behavioural dissociation between the consistent ability to predict targets and the exacerbated sensitivity to distraction in older age. Additionally, our findings revealed that the benefits of spatiotemporal predictions scale with the perceptual demands on visual search. When facing a low distraction, predictions do not provide a behavioural advantage even though the spatiotemporal regularities remained the same throughout the experiment irrespective of visual distraction load.

We previously compared performance in a dynamic visual-search task across the adult lifespan^22^ under a fixed amount of distraction. In our previous study, we also found preservation of benefits of spatiotemporal predictions despite a systematic age-related decline in performance overall. In our current study, we find that older adults can leverage their experience with spatiotemporal regularities to enhance performance independently of the degree of competition from distractors. This is an important extension of the previous work, supporting the independence of attentional guidance that is driven by predictions and learning vs. goal-driven attention under the traditional manipulation of distractor competition.

Our findings support and extend the broader literature on static visual search. Here, we provide the first evidence for a search slope within a dynamic search task. As shown in previous works using static tasks, the search slope can be manipulated by adding visual distractors^3^. We identified comparable effects in a dynamic and extended context. As seen in previous works about cognitive ageing, adding targets impaired performance, leading to a greater search slope in the group of the older adults^32,33^. Uniquely, we observed the same pattern in the continuous, extended context of the Dynamic Visual Search.

Changes in performance with increasing age also echo those found in static search tasks. Performance declined in older age^11^. Aging also exacerbated the negative impact of distraction^14,15,34^. Yet, in agreement with the developmental literature, age did not compromise the ability to use experience to enhance search performance. Similar evidence is found in studies involving contextual cueing^35–37^ and expectations about features and locations of targets^23,24^.

Contemporary neuroscientific models of lifespan development suggest that, as age increases, neural networks supporting cognitive control exhibit heightened activation and often engage broader regions^38^. A central tenet of these models is the dynamic compensation for declines in fluid abilities, such as response speed, problem solving, and working memory^38–40^. For instance, the Compensation-Related Utilization of Neural Circuits Hypothesis (CRUNCH^41^ proposes that older adults compensate for reduced efficiency in cognitive tasks by recruiting additional cortical regions, thereby utilizing more cognitive resources. This concept of increased resource utilisation is also reflected in other frameworks, such as the Hemispheric Asymmetry Reduction in Older Adults (HAROLD^42^model. HAROLD specifically addresses the tendency of the aging brain to show less lateralised task-related activation patterns compared to younger adults, attributed to the engagement of compensatory mechanisms. Similarly, the Scaffolding Theory of Aging and Cognition (STAC^43^; posits that individuals develop compensatory neural scaffolding to maintain cognitive performance amidst age-related changes. Although our study did not include direct neural measurements, the observed compensatory patterns align with these theoretical frameworks. Specifically, while target detection—a function closely linked to fluid abilities^44^—declined substantially with age, older participants effectively utilised predictive strategies to meet escalating task demands. It is plausible that this compensation was underpinned by increased neural activity compensating for diminished top-down and sensory signals. Future studies can adapt our experimental design to establish a connection between prediction-driven guidance and the observed patterns of compensatory overactivation in neural activity.

The preserved ability of older adults to learn and then utilise predictions in complex tasks, even amid visual distractors, is particularly significant. This finding suggests that the mechanism enabling the guidance of attention based on learning operates independently of goal-driven attention control. Previous theoretical work has distinguished between prediction-based and top-down attentional guidance^45–47^. Our study provides developmental evidence for these distinct sources of attentional guidance. Despite older adults exhibiting reduced goal-based target-identification performance and increased sensitivity to the number of distractors, they demonstrated behavioural benefits from predictions comparable to those of young adults. This preserved ability should be considered a crucial mechanism in aging research, as it supports behavioural performance despite other declining cognitive abilities.

Future studies could further investigate the distraction manipulation to identify the conditions under which prediction benefits are sustained. For example, distraction could be varied by amplifying background noise, introducing salient yet irrelevant information, or increasing the number of distractors. Such investigations could help clarify whether prediction-driven guidance is specifically tied to goal-directed behaviour, as influenced by an increased number of distractors, or whether it can also enhance performance in the presence of heightened bottom-up noise. Another interesting direction to deepen our understanding of prediction-driven guidance in aging is to investigate the level of explicit awareness regarding the predictability manipulation. The current study did not include a debriefing session to assess whether participants were aware of the embedded target regularities. Previous research has demonstrated that older adults can perform comparably to younger adults on tasks relying on implicit learning, whereas performance tends to decline when explicit awareness is required^48,49^. Although the prediction manipulation in our study was entirely implicit, as it was not the primary goal of the task, it is possible that some participants were aware of the manipulation, which may have differentially influenced their performance.

Despite the consistent benefits of spatiotemporal predictions across age, these regularities were used only in contexts where the number of distractors was high (i.e., 24). This pattern was observed in both age groups. It is worth noting that our previous studies using dynamic visual search included a distractor load that was higher than our low number of distractors condition (previously we used between 20 and 40 distractors in various designs and contexts, here we use 12). Therefore our previous results so far have shown statistically significant benefits of spatiotemporal predictions^27,28,30^. However, the link between increased distraction and reliance on predictions was observed in a different context using dynamic visual search. Previously, we identified a significant connection between prediction formation and the temporal proximity of two sequential targets^22^. When two targets appeared close in time, predictions were much more pronounced compared to when they were separated by longer intervals. These data, along with our current observations, indicate that attention may be more biased towards predictable signals in contexts where there is increased distraction.

In summary, we illustrate how observers implicitly gather information and learn where and when to focus based on task-related patterns. They then use these predictions when the distraction load increases. The manipulation of distraction also allowed us to replicate traditional findings in visual search (i.e., search slope) in a dynamic context that is more akin to natural environments. Notably, these findings were observed in an online sample, where control over factors that could influence perception, such as hardware variability and perceived stimulus size, was minimal. In our view, the ability to detect the differential effects of predictions and goal-driven guidance under these inherently noisier conditions lends further validity to our results, as they remained significant despite the limitations of online testing compared to laboratory settings. Nonetheless, it would be valuable to explore similar designs under more controlled conditions, particularly ensuring equal stimulus presentation sizes, to determine whether more nuanced effects emerge when sensory input is more precisely standardised. Alternatively, future replications could rely on relevant development that were validated to improve the control of viewing distance in online experiments^50^.

From a developmental perspective, our findings provide an important insight about attention control in older age. Experience-driven guidance can help mitigate age-related performance decline. Notably, the pattern of using predictions remained consistent across age differences, despite including two highly distinct age groups that otherwise differ significantly in their attention abilities.

## Methods

All experimental procedures and protocols were reviewed and approved by the University of Oxford Central University Research Ethics Committee. All research was performed in accordance with relevant research guidelines.

### Participants

We used Prolific, a participant recruiting platform for online experiments^51^. We included participants who were the relevant age (younger adults aged 18 to 22, and older adults aged 68 to 72), had normal or corrected-to-normal vision, no diagnosed psychiatric or neurological condition, a minimum of ten completed studies on Prolific, and an approval rating of at least 75% (percentage of Prolific studies for which the participant’s data had been approved). All participants used a personal computer to complete the study (i.e., the experiment did not support phones or tablets).

Our previous studies with younger adults indicated that a sample size of 25 provided strong statistical power, exceeding 0.9, to observe the benefits of spatiotemporal predictions in in-person studies. The results from these power analyses can be found in OSF (DOI 10.17605/OSF.IO/FP3SV). In this study, we also introduced a second manipulation of distraction, i.e., the number of distractors. Furthermore, we used an online platform and included older adults in the study. Each of these modifications could decrease power and introduce noise. Therefore, we aimed to collect a significantly larger sample of 120 individuals from each age group. This sample size was based on our previous in-person studies with young children aged 5–6 years, in which we sampled 80 individuals. Given the conservative assumption that effect sizes in behavioural experiments may be overestimated, we increased the sample size by 50%.

We recruited 258 participants to reach the target of 240 data sets (120 per group) of sufficient quality group for analysis. Data from 18 participants did not reach the inclusion criteria (see below) and were replaced. The resulting cohort is described in Table 1, where we report the mean Age, Sex and years of education (which did not differ between the two groups; t(159.51) = 1.32; *p* =.19). Participants provided informed consent and were compensated for their time at £8 per hour, in line with the recommended payment made by Prolific at the time of data collection (2021–2022).

**Table 1:** Descriptive statistics. Education is noted in years, based on individual assessment of years spent in school, higher education and professional training.

Group	Sex	Age	Education
Young adults	64 M; 54 F; 2 N/A	20.9 (SD = 1.7)	15.13 (SD = 2.1)
Older adults	54 M; 65 M; 1 N/A	69.7 (SD = 1.5)	15.78 (SD = 4.9)

### Apparatus

The experimental task was generated using PsychoPy^52^ and hosted on Pavlovia (http://pavlovia.org). Briefings were carried out using Qualtrics (http://qualtrics.com).

### General procedure

Participants were first presented with the information sheet and then provided informed consent. They filled in basic demographic data (e.g., age and sex) and then completed the *Dynamic Visual Search* Task (see details below). The session lasted approximately 25 min.

### Dynamic visual search task

#### Stimuli

**Fig. 2:**
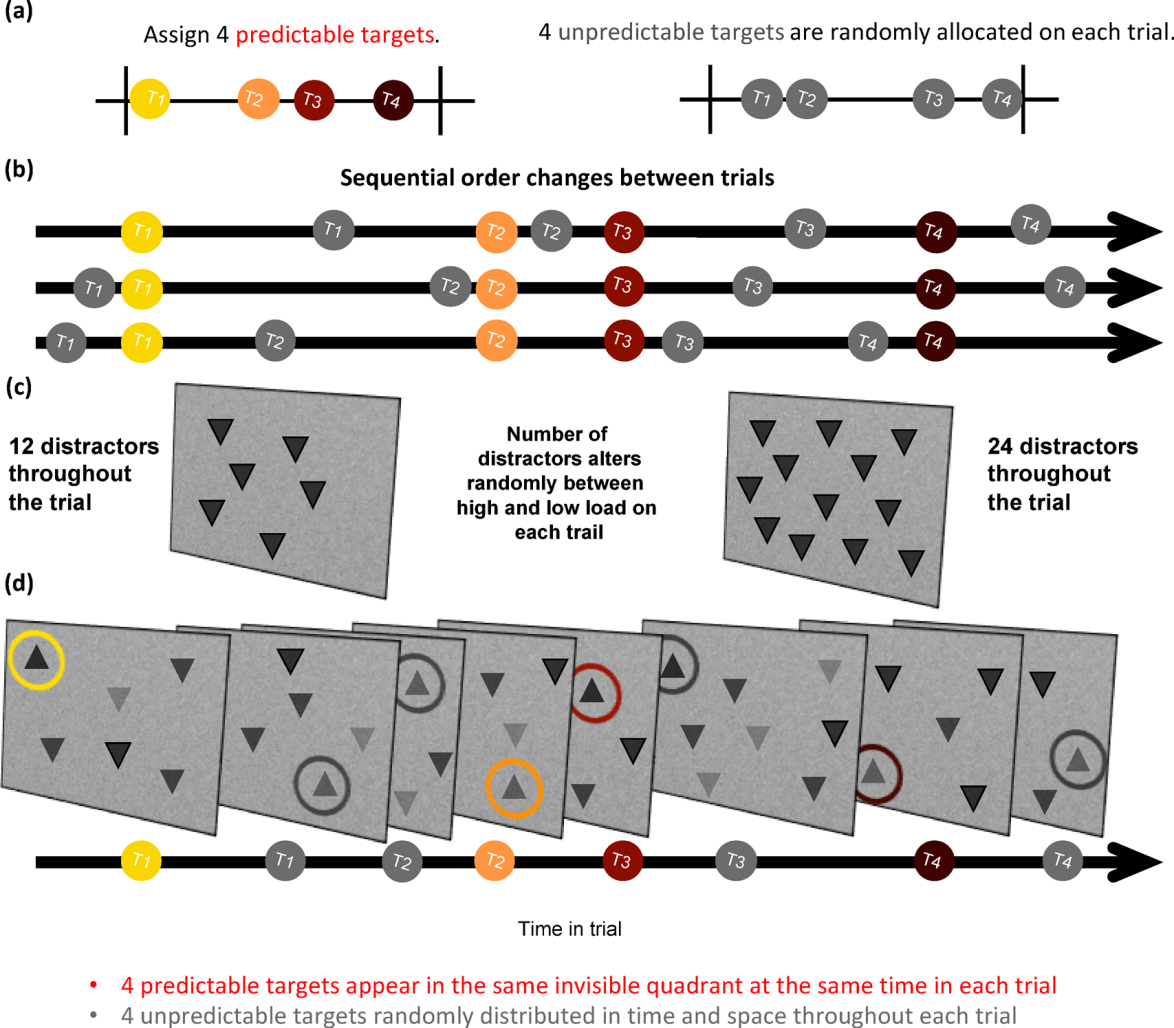
Illustration of the Dynamic Search Task. **(A)** Each trial was divided into four time bins. Four predictable targets appeared in each trial, each pre-assigned to one of the four bins. The other four unpredictable targets were each assigned randomly to a time bin and were jittered. **(B) **The coloured dots represent predictable targets over time, which always occurred at the same time-point within their assigned bin across a block of trials. Note that the colours are only used for illustration purposes. In the experiments, all the stimuli appeared in grey. The grey dots represent the four random targets, which were randomly distributed over time.** (C)** The total number of visual distractors that appeared on each trial varied between two conditions: high-distraction load (24 distractors) and low-distraction load (12 distractors). **(D) **The four predictable targets were assigned to four different quadrants that were kept constant throughout a block of trials. The quadrants for random targets were randomly determined. An example of a single experimental trial can be found on our OSF page (DOI: 10.17605/OSF.IO/8UPY5).

Figure 2 shows the task. Participants were instructed to find eight upward-pointing triangles (‘targets’) on each trial and ignore downward-pointing triangles (‘distractors’). Participants were given no information about the possible predictability of certain targets. Trials lasted approximately fourteen seconds and consisted of the eight targets and either twelve or twenty-four distractors fading in and out of the search display over its duration. The number of distractors was determined pseudorandomly between trials, with an equal number of trials with high- and low-distraction loads.

The search display had a static white-noise background. Target and distractor stimuli were black. Stimuli faded in and out of view slowly but did not move. Fade-in time was one second (gradually becoming visible until reaching a maximum of 80% opacity). The target stayed on the screen for another second and then faded out over another second. Each stimulus had a size of 0.07 normalised units (length and width), with 1.0 being the full-screen size. Stimuli could appear in any location on the screen but not overlap with another. Whenever participants identified a target, they used their mouse or trackpad to click on its location.

#### Spatiotemporal expectations

Of the eight target stimuli, four were spatially and temporally predictable (Predictable targets). On every trial, they appeared at the same temporal onset from the start of the trial and within the same quadrant of the full screen - top left, bottom left, top right, or bottom right. Location within the quadrant remained variable. The onset times for predictable targets were pre-assigned as follows: we created twenty-four equally spaced time points between the earliest (0.6 s) and latest (10 s) possible onsets. The interval between the time points was approximately 0.41 s. The resulting linearly spaced segments were further split into four equal ‘bins’, each consisting of six equally spread onsets.

To select the four predictable onsets for a given participant, one onset was drawn from each of the four time bins at the beginning of the experiment. Each of these four onsets was then randomly assigned to a different spatial quadrant. The onsets and their associated spatial quadrants then remained fixed for that participant throughout the experiment. Different participants had different interval-quadrant pairings for predictable targets.

The four remaining targets appeared at unpredictable quadrants and temporal onsets (Variable targets). Their timing was distributed pseudorandomly by assigning each to a random onset within a different time bin, ensuring that each of the four time bins presented one predictable and one unpredictable target while preventing overlap. To increase the overall temporal variability for stimulus appearance across trials, the exact onset times for each variable target were further jittered by adding or subtracting a value between 0 and 500 ms. These constraints ensured that targets were roughly evenly distributed throughout the trial and prevented too many target events from occurring at one time. All remaining unused onsets from the distribution were used for distractors with the same jittering approach. The locations of variable targets and distractors were chosen randomly at the beginning of each trial and were unique (i.e., different stimuli never appeared in the same location within a trial).

#### Number of distractors

In each trial, participants were instructed to search for eight visual targets (upward-pointing triangles) and ignore visual distractors (downward-pointing triangles). Between trials, we manipulated the number of visual distractors that could appear. The number of distractors altered between twelve and twenty-four, introducing varying levels of difficulties. The order of low/high distraction trials was assigned pseudorandomly, maintaining an equal number of trials in each condition. Visual distractors faded in and out with the same pace and spatial distribution as the visual targets. They never overlapped in spatial position.

#### Experimental procedure

Participants completed ten practice trials to become familiar with the task. They then completed a single block of 40 experimental trials, in which a total of 320 targets could be detected. During practice, all targets appeared at random times and locations (i.e., there were no ‘predictable’ targets). Each practice trial contained 8 targets and 12 distractors. In experimental trials, 4 targets appeared predictably and 4 appeared unpredictably among 12 or 24 unpredictable distractors. Participants were not informed about the predictability or distractor manipulations. Overall, there were 160 predictable and 160 variable targets per participant (excluding practice trials). Of these, half of the targets were presented during trials 12 distractors and half were presented during trials with 24 distractors. After each trial, observers received feedback indicating how many targets they found and how many trials remained until the end of the experiment. The experimental task lasted approximately 20 min.

### Analysis

Behavioural data were analysed using R (R Core Team, 2018). Inclusion criteria for data analysis required individuals to identify more than 35% of targets overall (across predictable and variable categories). The threshold was based on the detection of behavioural outliers with mean performance outside 1.5 times the interquartile range above the upper quartile and below the lower quartile. All the outliers identified performed below the lower threshold. Based on the exclusion criteria, we removed twelve participants. In addition, we removed six additional participants who did not complete the task. Altogether, 18 participants were removed and replaced by new participants of the same age group. Analysis of the included data examined the influence of target predictability, number of distractors, and age on performance.

A mixed-effects ANOVA was used and followed by appropriate *post-hoc* analyses using the emmeans function in R^53^ when appropriate. The emmeans (estimated marginal means) package in R is a statistical tool used to compute and compare model-based means (marginal means) by accounting for all factors and covariates in the fitted statistical model. For post-hoc tests, emmeans calculates these adjusted means for specific factor levels or combinations and conducts pairwise comparisons or custom contrasts to identify significant differences. Unlike raw-data-based approaches, emmeans offers several advantages: (1) it incorporates the structure of the model (e.g., interactions, covariates, and random effects), ensuring comparisons are aligned with the fitted analysis; (2) it handles unbalanced designs and repeated measures by using the proper error structure; and (3) it offers clarity through results that are model-adjusted and interpretable. These features make emmeans a robust and reliable tool for post-hoc analysis in complex experimental designs.

#### Dependent variables

The primary dependent variable for analysis purposes was the target hit rate. In our previous work, hit rates have proved to be a reliable and sensitive measure of the benefits of spatiotemporal predictability in children, young adults, and older adults^22,26–28,30,31^. Focusing on accuracy reduced the potential influence of age-related differences in response speed.

For completion, and to test for possible speed-accuracy trade-offs, we conducted secondary analyses with the standardised reaction times and the Balanced Integration Score as the dependent variable (BIS)^54^. When analysing reaction time data, we first remove extreme values exceeding three standard deviations from the mean for each participant. Subsequently, we analysed the z scores as our dependent variable. The BIS is a measure that controls for speed-accuracy trade-offs by equally weighting reaction times and hit rates. It is calculated by standardising reaction times and hit rates, and then subtracting the standardised hit rates from the standardised reaction times.

## Data Availability

Data are available in OSF: https://osf.io/35tq4/?view_only=2d05465f3a474e4d996380779971155e.
